# Secondary Failure of Tocilizumab in Treating Elderly-Onset Rheumatoid Arthritis With Systemic Symptoms Complicated by Diverticulum Perforation

**DOI:** 10.7759/cureus.28357

**Published:** 2022-08-24

**Authors:** Soshi Takagi, Yumi Naito, Chiaki Sano, Ryuichi Ohta

**Affiliations:** 1 Family Medicine, Shimane University Medical School, Izumo, JPN; 2 Communiy Care, Unnan City Hospital, Unnan, JPN; 3 Community Medicine Management, Shimane University Faculty of Medicine, Izumo, JPN

**Keywords:** rural hospital, general physician, anti-nuclear antibody, isoniazid, drug-induced lupus, tocilizumab, crp, diverticulum perforation, secondary failure of tocilizumab, elderly-onset rheumatoid arthritis

## Abstract

The treatment of rheumatoid arthritis (RA) has advanced from the use of steroids to disease-modifying anti-rheumatic drugs (DMARDs) and biologics such as tumor necrosis factor (TNF) and interleukin-6 (IL-6) inhibitors. Historically, steroids have been the mainstream in the clinical treatment of RA; however, the development of DMARDs has changed the RA treatment structure. In addition, biologics can alleviate RA symptoms. This case report describes the secondary failure of tocilizumab in treating RA with fatigue symptoms. Treatment with tocilizumab decreases C-reactive protein (CRP) levels, which may make detecting RA exacerbation difficult; therefore, obtaining the patient's precise history and thorough physical examinations are necessary. This case demonstrates the complexity of treating elderly-onset RA and reports practical methods for effective treatment.

## Introduction

The treatment of rheumatoid arthritis (RA) has advanced from the use of steroids to disease-modifying anti-rheumatic drugs (DMARDs) and biologics such as tumor necrosis factor (TNF) and interleukin-6 (IL-6) inhibitors [1,2]. Steroids have been the mainstream treatment in the clinical history of RA therapy; however, the development of DMARDs has changed the RA treatment structure [3]. DMARDs, such as methotrexate (MTX), have now become the mainstream treatment for RA [4]. In addition, biologics are also used to alleviate RA symptoms [4]. The introduction of DMARDs and biologics has improved patients' quality of life with RA [1,2]. Therefore, with the increasing advances in the treatment of RA, these must be updated in rural hospitals.

The increase in the use of biologics could confuse the treatment of RA because biologics can influence changes in the biomarkers of inflammation. For instance, anti-IL 6 inhibitors can induce a drastic decrease in the amount of C-reactive protein (CRP) in the serum, which is a clinical indicator of inflammation in RA [1,2]. Moreover, the continuous use of biologics may cause secondary failure due to the production of antibodies against the biologics. The human body can produce antibodies against biologics, rendering biologics ineffective in treating patients with RA [1,2]. Additionally, the secondary failure rate may increase in patients with RA who do not use DMARDs. Therefore, CRP should not be used to detect disease exacerbation in RA patients using anti-IL 6 inhibitors [4]. Here, we report the case of an older woman diagnosed with elderly-onset RA (EORA) treated with tocilizumab, with chief complaints of fatigue and systemic pain. In this case, we could not use CRP to detect RA exacerbation, which necessitated obtaining a precise medical history and thorough physical examination. Herein, we discuss the complexity of treating EORA and report practical methods for effective treatment.

## Case presentation

A 72-year-old woman presented to our hospital with chest tightness, dizziness, and abdominal fullness. She had visited our ER with the same symptoms five days prior. At that time, the patient was diagnosed with benign paroxysmal positional vertigo (BPPV). Four days after the BPPV diagnosis, she visited our otorhinolaryngology department, and the doctor followed up on her symptoms. Her medical history included EORA, osteoporosis, spinal canal stenosis, cataracts, and vertebral compression fracture. Her medication history included tacrolimus, trimethoprim-sulfamethoxazole, tocilizumab, esomeprazole, and risedronate. For her EORA, she had been using prednisolone for three years until six months prior, when prednisolone was switched to tacrolimus and tocilizumab. She had last used tocilizumab one week before the visit.

Her vital signs were as follows: blood pressure of 148/78 mmHg, heart rate of 81/min, body temperature of 36.6° C, respiratory rate of 18/min, and oxygen saturation (SpO_2_) of 97% on room air. Her consciousness was clear. Physical examination revealed right lower abdominal tenderness upon percussion. Laboratory tests revealed normal white blood cell count and C-reactive protein levels (Table 1).

**Table 1:** Initial laboratory data of the patient CK - creatine kinase; CRP - C-reactive protein; TSH - thyroid-stimulating hormone; HCV - hepatitis C virus; SARS-CoV-2 - severe acute respiratory syndrome coronavirus 2; HBs - hepatitis B surface antigen; HBc - hepatitis B core antigen; C3 - complement component 3; C4 - complement component 4; MPO-ANCA - myeloperoxidase antibody proteinase 3 antibody; SS - Sjögren's syndrome; CCP - cyclic citrullinated peptide

Marker	Level	Reference
White blood cells	3.7	3.5–9.1 × 10^3^/μL
Neutrophils	55.2	44.0–72.0%
Lymphocytes	35.3	18.0–59.0%
Monocytes	7.8	0.0–12.0%
Eosinophils	1.3	0.0–10.0%
Basophils	0.4	0.0–3.0%
Red blood cells	3.70	3.76–5.50 × 10^6^/μL
Hemoglobin	12.2	11.3–15.2 g/dL
Hematocrit	35.9	33.4–44.9%
Mean corpuscular volume	97	79.0–100.0 fl
Platelets	18.3	13.0–36.9 × 10^4^/μL
Erythrocyte sedimentation rate	10	2–10 mm/hour
Total protein	6.7	6.5–8.3 g/dL
Albumin	3.4	3.8–5.3 g/dL
Total bilirubin	0.8	0.2–1.2 mg/dL
Aspartate aminotransferase	53	8–38 IU/L
Alanine aminotransferase	20	4–43 IU/L
Alkaline phosphatase	156	106–322 U/L
γ-Glutamyl transpeptidase	269	<48 IU/L
Lactate dehydrogenase	161	121–245 U/L
Blood urea nitrogen	18.9	8–20 mg/dL
Creatinine	0.71	0.40–1.10 mg/dL
Serum Na	137	135–150 mEq/L
Serum K	3.9	3.5–5.3 mEq/L
Serum Cl	102	98–110 mEq/L
CK	124	56–244 U/L
CRP	0.04	<0.30 mg/dL
TSH	0.34	0.35–4.94 μIU/mL
Free T4	1	0.70–1.48 ng/dL
HBs antigen	0.0	IU/mL
HBs antibody	0.67	mIU/mL
HBc antibody	0.00	S/CO
HCV antibody	0.00	S/CO
Syphilis treponema antibody	0.00	S/CO
SARS-CoV-2 antigen	Negative	
anti-nuclear antibody	320	<40
homogeneous	320	<40
C3	55	86–164 mg/dl
C4	5	17–45 mg/dl
MPO-ANCA	<1.0	<3.5 U/ml
anti-SS-A/Ro antibody	<1.0	<10.0 U/ml
anti-SS-B/La antibody	<1.0	<10.0 U/ml
anti-CCP antibody	<0.6	<5 U/ml
Urine test		
Leukocyte	Negative	
Nitrite	Negative	
Protein	Negative	
Glucose	Negative	
Urobilinogen	Normal	
Bilirubin	Negative	
Ketone	(3+)	
Blood	Negative	
pH	5	
Specific gravity	1.018	

**Figure 1:**
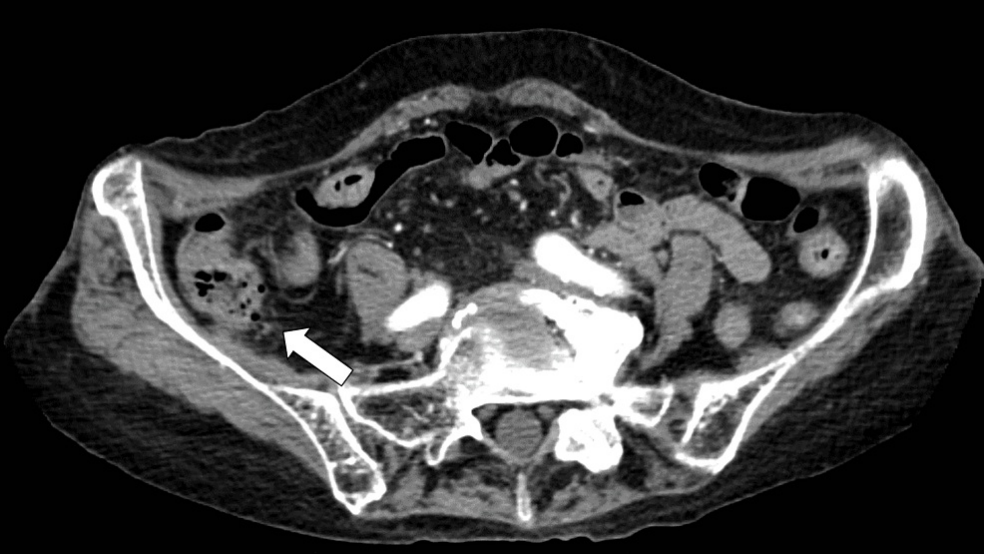
Enhanced computed tomography showing multiple diverticula with extra-luminal gas in the ascending colon

Contrast-enhanced abdominal computed tomography (CT) revealed multiple diverticula with extraluminal gas in the ascending colon (Figure 1).

Therefore, diverticulitis or perforation of the diverticulum was suspected. Hence the patient was treated with fasting and tazobactam/piperacillin.

After admission, the patient developed pain in both shoulders and thighs, which exacerbated. Additionally, shoulder synovitis and bursitis were observed on ultrasound. We inferred that these symptoms were caused by RA exacerbation due to secondary failure of tocilizumab and started prednisolone 15 mg/day. We also considered drug-induced lupus caused by isoniazid as another possible cause of the symptoms because the antibodies (homogenous and speckled) levels were >320 times. The results of the tests for anti-dsDNA, anti-RNP and anti-Sm antibodies were all negative; hence, the anti-histone antibody was suspected to be a specific autoantibody. Given that there were no hematological abnormalities or other dermal or serosal inflammatory findings, her condition did not meet the diagnostic criteria for drug-induced lupus. Moreover, because the T-SPOT® test was negative and the possibility of latent tuberculosis infection was low, isoniazid was discontinued.

The shoulder and thigh pain improved on the fourth day of hospitalization and disappeared on the eighth day. The prednisolone dose was reduced to 10 mg, and 6 mg of MTX was added to her treatment. The patient was discharged on the 15^th^ day of hospitalization. In the outpatient follow-up, prednisolone was gradually tapered, and methotrexate was increased to 12 mg/week.

## Discussion

Tocilizumab is an antibody against the IL-6 receptor used when RA symptoms are difficult to control with DMARDs. However, suppressing immunity by suppressing IL-6 can make detecting serious infections or RA exacerbations difficult. In particular, the decrease in CRP levels caused by IL-6 inhibition may delay the detection of RA exacerbations due to secondary failure.

Intestinal perforation when using tocilizumab should be noted among infectious diseases because it can be fatal [5]. Although the mechanism of intestinal perforation by tocilizumab has not been clarified, tocilizumab suppresses Th17 cells, which play an important role in the biological defense against infection through innate and acquired immunity in the intestinal mucosa [6]. Specifically, 10-30% of older people have colon diverticulum and are anatomically vulnerable; therefore, they often have diverticulum perforation while using tocilizumab [7]. In cases of RA refractory to MTX, tocilizumab must be used for disease control [4]. Thus, there is a need for risk assessment of the complications associated with tocilizumab and follow-up of the symptoms related to complications such as diverticulum perforation.

Secondary failure of tocilizumab is detected through precise clinical history and physical examination and is not based on laboratory findings. Approximately 1.5% of patients with RA treated with subcutaneous tocilizumab develop an anti-drug antibody, which causes secondary failure of tocilizumab. When treating EORA with tocilizumab, MTX should be concomitantly used to prevent secondary failure [8,9]. However, older patients often have chest radiography or CT abnormalities, which require them to avoid using MTX [10,11]. For instance, in the reported case, the local stricture of the bilateral lungs prevented the use of MTX. There is negative evidence regarding the exacerbation of interstitial pneumonia with low doses of MTX, although this evidence is inconclusive [10,11]. However, given tocilizumab can cause various complications. RA should be treated with MTX when patients have no clear interstitial shadow or an increase in KL-6. 

Considering the possibility of secondary failure and complications associated with serious infections is necessary when tocilizumab is used. In this case, the patient had indefinite complaints such as chest tightness, dizziness, and abdominal fullness. Based on the patient's clinical course, we suspected diverticulitis and perforation of the diverticulum and treated the patient with antibacterial agents. We considered the possibility of secondary failure of tocilizumab because of the general muscular and joint pain and tenderness. Therefore, tocilizumab was discontinued, and treatment with prednisolone and MTX was initiated, which proved effective. Secondary failure can be rare but happen without the usage of MTX, so the possibility should be considered in the treatment of EORA.

Medications can cause various side effects, such as joint pain, and can facilitate the production of autoantibodies, making it difficult to distinguish these side effects from collagen diseases. In this case, isoniazid was suspected as a possible cause because joint pain manifestation was similar to drug-induced lupus. In the tocilizumab treatment course, latent tuberculosis infection (LTBI) is a feared infectious disease. Treatment intervention is recommended for LTBI patients taking prednisolone or immunosuppressants because of the increased risk of active tuberculosis [12]. Although isoniazid is included in the treatment regimen for LTBI, it has a moderate risk of drug-induced lupus erythematosus (DILE). The mechanism of DILE is unknown, but symptoms such as joint pain, muscle aches, fever, serositis, and rash generally appear within a few months to a few years of drug administration [13]. Typically, the results for anti-nuclear and anti-histone antibodies are positive, and those for specific antibodies (anti-Ds-DNA and anti-Sm antibodies) are negative. In our case, isoniazid was not used to activate the LTBI. Since the results for all the specific antibodies were negative, the anti-histone antibody was speculated to be an anti-nuclear antibody. This suggested the possibility of drug-induced lupus caused by isoniazid. Thus, after confirming that the T-SPOT® test was negative, isoniazid was discontinued.

When older patients with RA complain of general pain and arthralgia, it is necessary to consider various possibilities, such as RA exacerbation, secondary failure, or other complications. However, it is difficult to distinguish between these conditions because they have similar clinical symptoms in older patients. Moreover, older patients tend to have vague symptoms, leading to different and incorrect help-seeking behaviors, which can exacerbate their symptoms [14,15]. As a result, medical professionals deal with their symptoms in a mild and undeferential manner, with symptomatic treatment affected by ageism [16,17]. In particular, various symptoms often appear because of autoimmunity in older patients with autoimmune diseases such as RA [18,19]. Therefore, we strongly suggest considering various possibilities of RA when symptoms of older patients with RA worsen; to this end, health care professionals should take accurate clinical histories, perform precise physical examinations, and follow up with the patients closely.

## Conclusions

Here, we report a case of EORA with possible secondary failure of tocilizumab in a community hospital. The characteristics of tocilizumab, the patient's age, and various medicines used to prevent complications complicated this case. Since tocilizumab can cause various complications and secondary failure, clinicians should take precise clinical histories, perform thorough physical examinations, and use MTX appropriately by considering the risk of interstitial pneumonia. Furthermore, medical professionals should deal with older patients' symptoms without ageism, especially in patients with autoimmune diseases.
